# Behavioural regulation of mineral salt intake in honeybees: a self-selection approach

**DOI:** 10.1098/rstb.2021.0169

**Published:** 2022-06-20

**Authors:** Raquel T. de Sousa, Robyn Darnell, Geraldine A. Wright

**Affiliations:** ^1^ John Krebs Field Station, Department of Zoology, University of Oxford, Oxford OX2 8QJ, UK; ^2^ Institute of Neuroscience, Newcastle University, Newcastle upon Tyne NE1 7RU, UK

**Keywords:** *Apis mellifera*, gustation, feeding, Bertrand's rule, nutrition, minerals

## Abstract

Minerals are required in small amounts to sustain metabolic activity in animals, but mineral deficiencies can also lead to metabolic bottlenecks and mineral excesses can induce toxicity. For these reasons, we could reasonably expect that micronutrients are actively regulated around nutritional optima. Honeybees have co-evolved with flowering plants such that their main sources of nutrients are floral pollen and nectar. Like other insects, honeybees balance their intake of multiple macronutrients during food consumption using a combination of pre- and post-ingestive mechanisms. How they regulate their intake of micronutrients using these mechanisms has rarely been studied. Using two-choice feeding assays, we tested whether caged and broodless young workers preferred solutions containing individual salts (NaCl, KCl, CaCl_2_, MgCl_2_) or metals (FeCl_3_, CuCl_2_, ZnCl_2_, MnCl_2_) in a concentration-dependent manner. We found that young adult workers could only self-select and optimize their dietary intake around specific concentrations of sodium, iron and copper. Bees largely avoided high concentration mineral solutions to minimize toxicity. These experiments demonstrate the limits of the regulation of intake of micronutrients in honeybees. This is the first study to compare this form of behaviour in one organism for eight different micronutrients.

This article is part of the theme issue ‘Natural processes influencing pollinator health: from chemistry to landscapes’.

## 1. Introduction

Honeybees live in densely populated colonies with overlapping generations [1], which means they are constantly striving to meet the colony's demand for nutritional resources. One adaptation within a honeybee colony is the division of labour among adult workers. Adult foragers select and collect pollen and nectar from flowers. Collected pollen is stored in the colony and consumed by relatively young adult workers, who process food to feed developing larvae and reproducing bees. By consuming the food and creating glandular secretions, adult workers regulate the intake of nutrients for the entire colony [2,3].

Bee nutrition is partitioned into macronutrient sources within a colony: honey is the primary source of carbohydrates, and bee bread (a mixture of pollen and honey) is the primary source of protein, fats, vitamins and minerals [4]. Pollen has a more diverse (K, P, S, Ca, Mg, Na, Cu, Fe, Mn, Zn) and a more concentrated mineral profile (2.5–6.5% total ash) than nectar or honey [5–7]. The mineral contents of pollen depend on soil and botanical origin, season and geographical location [8–10]. Like other herbivores, bees may be nutritionally deficient in sodium (Na) because they consume plant tissue [11]. Thus, one plant species' pollen may not fully complete the mineral needs of honeybees [8]. Collection of the pollen of many plant species may be the mechanism by which bees overcome nutritional deficiencies. Bees also consume honey, derived from floral nectar and other sources like aphid honeydew [12]). Honey is unlikely to provide all necessary micronutrients, as it has relatively low concentrations of minerals (total ash: 0.17–1%), with potassium (K) accounting for greater than 70% of the total ash content, followed by phosphorus (P), sulfur (S), chloride (Cl), sodium (Na) and sometimes magnesium (Mg) and other trace metals [5,7,13,14].

Insect nutrition is well studied for many species, including aphids, bees, flies and locusts [15–17]. Although micronutrient nutrition (e.g. minerals, vitamins) has been challenging to tackle and findings harder to generalize [18,19], we know that minerals are important for development, growth and fecundity in insects [15,19]. For example, dietary iron (Fe) or zinc (Zn) deficiencies retarded aphid larval growth from the first generation [19]. Minerals (salts and metals) are essential micronutrients performing structural, physiological, catalytical and regulatory functions within insect cells and tissues [20,21]. Sodium (Na) and potassium (K) (alongside chloride, Cl) are the most active ions within the body, and their balance is critical for osmoregulation [22], cold tolerance [23] and insects’ reproductive output [24,25]. Calcium (Ca) and magnesium (Mg) enable muscle contraction–relaxation and participate in intracellular communication and ATP metabolism, respectively [21]. In *Drosophila,* Ca is required for egg activation [26] and dietary Mg improves memory function [27]. Iron (Fe) is a component of several metalloenzymes involved in toxin degradation via cytochrome P450, moulting hormone production [28], innate immune response [29–31], magnetoreception in bees [32], fecundity in mosquitoes [33] and neurodevelopment in mammals [34]. Zinc (Zn) is a cofactor in many enzymes involved in the antioxidant defence against oxidative stress [35,36], metal detoxification mechanisms and cell replication [37]. Copper (Cu) and manganese (Mn) are essential cofactors in metalloproteins with catalytic activity (e.g. superoxide dismutase systems) [20,37]. However, they can occur in high concentrations in the soil and plant tissues and disrupt insect behaviour [38], particularly in pollinators [39,40]. These metals can also support the sclerotization and hardening of the insect cuticle [41,42].

Nutritional deficiencies can lead to changes in animal regulatory behaviour. For example, ants deficient in Na seek salt and prefer it over sugar baits [43]. Dietary minerals influence food perception and feeding decisions in locusts/grasshoppers [44,45], rats [46,47], fruit flies [48–51] and kissing bugs [52]; in addition, NaCl can act as a gustatory reinforcer in learning [48,49,53]. Previous work indicates that optimal ranges of micronutrients in bee diet exist. For example, pollen ash (1%) maximized brood rearing in bee colonies [54]. Others found that specific concentrations of dietary Ca improved larval growth and the antioxidant capacity of honeybees [55]. In *Osmia bicornis,* supplementation of larval food with Na, Zn and K increased female body mass, survival and male body mass, and cocoon development, respectively [56]. Honeybees faced with mineral limitations in pollen may forage on alternate food sources (e.g. water) [57–60] to (possibly) complement their nutrition. Other studies suggest that bees avoid ingesting high concentrations of minerals (e.g. high K) in food [61], which can downregulate the estimation of nectar profitability and foraging activity [62,63]. High metal concentrations in food negatively impact individual bees (e.g. health, cognition and foraging behaviour) [12,64–67], eventually inducing cumulative effects on the colony [39].

The extent to which bees regulate micronutrients in diet is largely unknown but could be revealed by studying food choice behaviour. Mineral salts are phagostimulant molecules that influence food palatability, with animals usually accepting low and rejecting high concentrations. In *Drosophila*, the ability to taste low Na and high Ca in food drives the appropriate feeding response which contributes to the regulation of ingestive behaviour [51,68]. Whether these behaviours generalize toward other mineral nutrients, however, is unclear. The relationship between deficiency and toxicity of micronutrients was formalized in a dose–response model referred to as the Bertrand's rule [69]. It predicts that animals will seek and ingest essential micronutrients in a concentration-dependent way; low levels are increasingly phagostimulatory (and beneficial for their health) until they reach an optimal plateau, whereas beyond that micronutrients can become toxic and are rejected [16,69,70]. We know micronutrients are required in small amounts but can become toxic at high levels. Though essential, Cu, Fe and Mn are redox-active metals that can induce oxidative stress and disrupt biological systems [71], and mineral nutrition can influence the antioxidant status of honeybees [36,55,71]. It is reasonable to expect that these compounds would have strict animal regulatory mechanisms. Some insects, like the aphid [19], the locust nymph [44] and the fruit fly larva [48–50], regulate mineral intake around preferred ranges, and termite colonies regulate their intake of multiple minerals in food [72]. By contrast, grasshoppers and field crickets do not appear to regulate the ingestion of dietary phosphorus [45,73]. Here, using two-choice feeding assays with caged and broodless young workers, we tested whether honeybees could self-select individual salts (NaCl, KCl, CaCl_2_, MgCl_2_,) and metals (FeCl_3_, CuCl_2_, ZnCl_2_, MnCl_2_) in food in a concentration-dependent manner. According to Bertrand's rule, we expect that honeybees show a preference for specific mineral diets if they actively and consistently consume more of the mineral compared with the control diet over time, and also that bees will choose a preferred and optimal range of concentrations if within or above their nutrient requirements (e.g. body contents) and below toxic concentrations, which negatively affect feeding and survival over time. Identifying the specific impacts of micronutrients in the feeding preferences that can lead to the optimization of intake by adult worker honeybees is, therefore, an important but neglected aspect of their nutritional ecology. This study is one of the first to assess dietary self-selection and the Bertrands' rule assumption on the ingestion behaviour of eight minerals individually tested in food. With this, we aimed to understand whether adult workers use taste to perceive and select preferred ranges, and regulate their intake of minerals in food.

## 2. Material and methods

### (a) Experimental animals and chemically defined diets

Young worker bees should best represent colony nutrient needs because they perform tasks within the hive that are tightly related to food processing and larval feeding [4]. Additionally, among adult workers and even when not tending for brood, they are the ones expected to have higher nutritional demands and to be more sensitive to micronutrients such as mineral salts.

Honeybee colonies (*Apis mellifera*, var. Buckfast) were kept in the northeast UK between May and September 2014–2016. Brood frames with capped cells were selected and marked from up to 30 colonies in the apiary. Within two days before the estimated eclosion (11–13 days after brood cell is capped), these marked frames were moved into a ventilated incubator (SANYO Electric Co., Japan) and kept at 34°C and 56% relative humidity in the dark [74]. To account for potential colony effects, emerging bees (0–30 h old) were brushed off different frames and mixed together into a large ventilated container before we assigned them to dietary treatments. We then randomly collected *n* = 30 bees from the container and assigned them to experimetal boxes in a random order. We did not feed newly emerged bees (new) between their emergence and assignment to dietary treatments (2–5 h). Therefore, we expected bees were more likely to be motivated to feed. At the end of the experiment, bees remaining alive were euthanized by freezing.

In laboratory conditions, broodless workers’ survival can be ensured by providing ad libitum sucrose solutions at 30–50% w/v [74]. Therefore, we used reagent grade sucrose dissolved in deionized water (pH ≈ 6.5) at 1.0 M (34.2% w/w) as the background solution to prepare mineral diets. We refer to minerals as both salts (macroelements) and metals (microelements). We tested the eight minerals most prevalent in pollen at five levels of concentration each. For salts, we used NaCl (0, 5, 50, 500, 1000 ppm), KCl (0, 10, 100, 1000, 10 000 ppm), CaCl_2_ (0, 1, 10, 50, 500 ppm) and MgCl_2_ (0, 10, 30, 300, 3000 ppm), and for metals we used FeCl_3_ (0, 1, 10, 100, 1000 ppm), CuCl_2_ (0, 0.5, 5, 50, 500 ppm), ZnCl_2_ (0, 0.5, 5, 50, 500 ppm) and MnCl_2_ (0, 1, 10, 50, 500 ppm); for reagent details refer to electronic supplementary material, S1. The range of concentrations for each mineral was based on recommendations on the mineral composition of synthetic diets for honeybees [54,75], and tailored for each mineral after preliminary work in our laboratory [76].

### (b) Experimental boxes and feeding tubes

**Figure 1.**
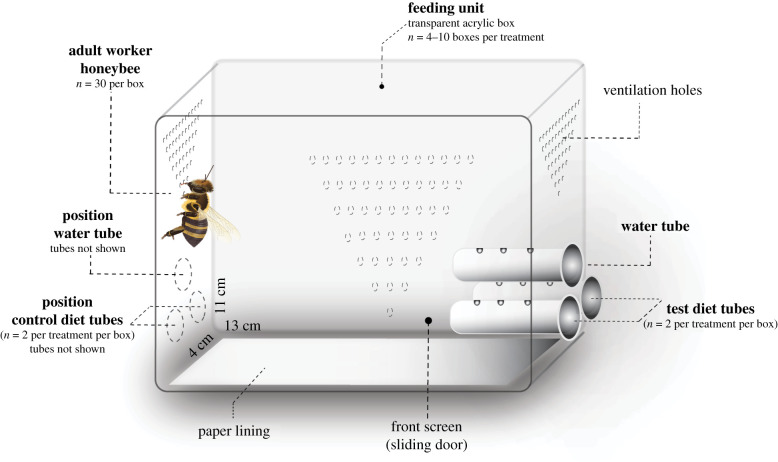
Diagram of experimental box and feeding tubes used in two-choice feeding assays (*n* = 30 bees per box). Control treatment boxes were fitted with four tubes of control diet (sucrose only) and two water tubes. For details see Material and methods. Adapted from [76].

Experimental boxes consisted of customized acrylic ventilated boxes (dimensions: 13 × 11 × 4 cm; 0.4 l capacity) with two sliding screens (front/back) and three holes (Ø 10.9 mm) in each side where feeding tubes were delivered (Bay Plastics, North Shields, UK) (figure 1). Each box was taken as one unit replicate and randomly assigned to diet treatments. Within each box, cohorts of *n* = 30 newly emerged workers (new) were able to move freely. Feeding tubes consisted of 2.0 ml microcentrifuge Eppendorf tubes (VWR International, UK) modified by drilling four holes (Ø 2.0 mm) in a line and 5 mm apart to allow lapping. Each box was fitted with six feeding tubes, i.e*.* one pair of both control and test diets (mineral treatments) or two pairs of control diet only (control treatment), and one pair of deionized water; for tube layout refer to electronic supplementary material, S2. We replaced feeding tubes with fresh diet every day; this hampers diet contamination by environmental dust, which could induce a build-up in trace elements or microbial growth [74].

### (c) Two-choice feeding assays and diet treatments

We used two-choice feeding assays in this study to investigate (1) dietary self-selection and non-randomness of food intake, (2) mineral preferences and how bees regulated the ingestion of individual minerals varying in concentration, and (3) the effects of mineral diets on the survival of young adult workers. The dietary context was as follows: honeybees were offered a choice between two diets: control diet (1.0 M sucrose solution) and a test diet (mineral-laced sucrose solution). We conducted a total of 40 diet treatments, each testing a single mineral diet at one out of five levels of concentration over 6 days (*n* = 4–10 boxes and *n* = 120−300 bees per treatment). Alongside each mineral group, we ran one control treatment (sucrose only, no mineral added) (*n* = 4–10 boxes). Owing to space limitations inside the incubator at certain times, we used fewer box replicates for some treatments (e.g. sucrose controls, iron, copper). In some instances, treatment boxes were knocked over and had to be discarded. Mock evaporation boxes were used to account for the evaporation loss of liquid diets. Each treatment was attended by *n* = 2–4 mock boxes (same tube setup without bees). All boxes were kept inside an acclimatized incubator (34°C and 56% relative humidity, no light) except when assessed. We shuffled the boxes inside the incubator every day to minimize shelf bias on the evaporation rates of diets [76]. We also monitored the temperature and relative humidity throughout the experiment (OMEGA Engineering, Manchester, UK).

### (d) Diet and water consumption, body weights and survival of young worker honeybees

For each experimental box, we weighed and replaced the feeding tubes (control, test solutions or water) every 24 h (±4 h) over 6 days. First, we estimated the differences in weight (g) to obtain consumption values for each diet. These values were adjusted by subtracting the mean percentage loss by evaporation for each diet. We measured evaporation rates for each solution in mock boxes containing the diets but without bees; these values were normalized for each solution—for details refer to electronic supplementary material, S3. Then, we determined the volume of each solution by dividing the weight of the consumed solution by its density (measured solution densities: 1.0–1.2 g ml^−1^). For each experimental box, the volumes consumed for each solution were divided by the number of live bees. Finally, we averaged the volumes consumed for each solution per bee over the duration of the experiment. Total consumption was a measure of the total volume consumed from both diet solutions and excluding water. Water intake was measured for each treatment, determined in a similar fashion, and analysed separately.

To better illustrate consumption patterns and feeding preferences for salt- or metal-laced sucrose diets, we calculated the preference index (PI) using the volumes consumed for each diet: PI =((test diet consumed) – (control diet consumed))/(total diet consumed). Preference indexes were estimated as total PI (mean over 6 days) or daily PI to assess how diet consumption and preferences fluctuated over time.

In a natural setting, bees defaecate while flying in the daylight. Here, although free to move inside the box, bees were mostly kept in the dark inside the incubator (except during assessment periods). Therefore, we further recorded fresh body weights at the end of experiment (endpoint) (*n* = 5 bees per box) to evaluate the impact of dietary treatments in caged workers. For qualitative comparison, we weighed untreated worker bees (no feeding treatment received) directly collected from brood frames (newly emerged bees; proxy of starting point) or at the entrance of the colony (fg, foragers).

Honeybee mortality was used as a proxy for health benefits/costs associated with active mineral ingestion. Therefore, we recorded the number of dead bees every day and removed them from the box. Control treatment (sucrose alone) was used as the reference treatment to compare survival curves between feeding groups for each mineral.

### (e) Statistical analyses

We performed statistical analyses using IBM SPSS Statistics for Macintosh (v. 27, 2022) and GraphPad Prism Software (Prism 9.3.1 for macOS, 2021). Total preference indexes and total volumes consumed were analysed using generalized linear models (GzLM). We first constructed a factorial model to test the effects of mineral type, concentration and the interaction between both factors on diet consumption. Then, we applied univariate GLzM models to each mineral group for *post hoc* comparisons between concentrations (control/reference group or pairwise) using sequential Bonferroni procedure. Within each treatment, we compared the volumes of control versus test solutions using unpaired *t*-tests with Holm–Šídák method (*α* = 0.05) for multiple comparisons. We analysed salts and metals separately. Diet consumption and water consumption were also analysed separately. The differences in daily preferences (daily PI) for each mineral treatment were analysed using generalized linear models estimating equations (logistic GEE) for repeated measures within-subjects. We built a factorial model to test the effects of time (days), concentration and the interaction between days and concentration. The impact of mineral diets on bee survival was estimated using survival analysis with Cox regression models and testing for the proportionality of hazards assumption, i.e. whether the risk factors affecting time to event (death) were constant over time. For each mineral, differences in survival across concentrations (risk factor) were compared using contrasts indicator with control treatment (sucrose alone) as the reference group.

## 3. Results

We offered caged young worker honeybees a choice between diets to investigate whether bees prefer a mineral-laced sucrose diet over sucrose alone (1.0 M aqueous solution). Here, we refer to minerals as salts (macroelements) or metals (microelements); for the salt group, we tested NaCl (Na, 5–1000 ppm), KCl (K, 10–10 000 ppm), CaCl_2_ (Ca, 1–500 ppm) and MgCl_2_ (Mg, 10–3000 ppm), and for the metal group we tested FeCl_3_ (Fe, 1–1000 ppm), CuCl_2_ (Cu, 0.5–500 ppm), ZnCl_2_ (Zn, 0.5–500 ppm) and MnCl_2_ (Mn, 1–500 ppm); control treatment (0 ppm) consisted of a single diet (1.0 M sucrose alone) with no mineral added to the sucrose solution. By measuring diet consumption over a range of concentrations tailored for each mineral, we were able to assess self-selection behaviour and mineral feeding preferences of young workers, and evaluate the impact of mineral feeding on their survival within the range of concentrations tested.

### (a) Mineral feeding preferences and diet consumption by caged young workers

#### (i) Salts

**Figure 2.**
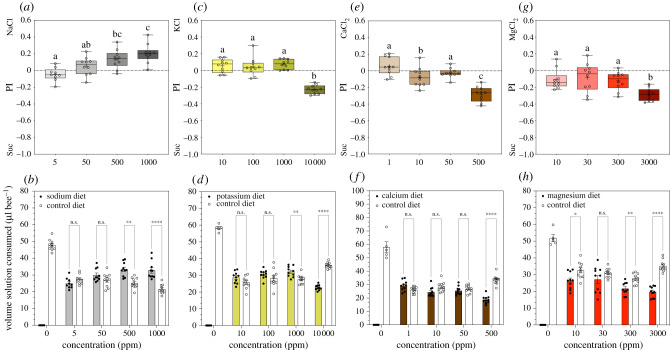
Consumption preference for salt-laced sucrose solutions in two-choice assays. (*a*,*b*) sodium, (*c*,*d*) potassium, (*e*,*f*) calcium and (*g*,*h*) magnesium treatments. (*a*,*c*,*e*,*g*) Boxplots refer to total consumption preference (PI) and indicate the minimum and maximum ranges for each independent mineral treatment; +, mean; −, median. Preference for the salt (PI > 0) or control (Suc, PI < 0) diets. We performed one-way GzLM to test the effects of concentration for each independent mineral group. Preference for salts: (*a*) Na: $x_{3}^{2}=40.4$, *p* < 0.001; (*c*) K: $x_{3}^{2}=208$, *p* < 0.001; (*e*) Ca: $x_{3}^{2}=81.7$, *p* < 0.001; (*g*) Mg: $x_{3}^{2}=31.5$, *p* < 0.001. Statistical differences between treatments are denoted by different letters (pairwise at *p*-value < 0.05). (*b*,*d*,*f*,*h*) Barplots indicate the volumes consumed from both solutions bees had access to (salt-laced and control diets); error bars represent the standard error of the mean (s.e.m.). We performed multiple comparison analysis to compare consumed volumes from both solutions at each level of mineral concentration; refer to electronic supplementary material, information S4. Asterisks denote statistical significance at *p < 0.05, **p < 0.01, ****p < 0.0001; n.s., not significant. Control treatment (sucrose-only diets, 0 ppm) was excluded from the pairwise comparison analysis. (Online version in colour.)

Feeding preferences and volumes consumed by young workers given salt-laced sucrose diets are indicated in figure 2. Overall, preference for salt diets depended on the salt identity and its concentration (total PI, GzLM: salt × concentration, $x_{9}^{2}=142$, *p* < 0.001). Similarly, and within each salt treatment, the volumes consumed from each diet (control or test) depended on the diet and salt concentration (figure 2*b*, Na: diet × concentration, $x_{3}^{2}=42.3$, *p* < 0.0001; figure 2*d*, K: diet × concentration, $x_{3}^{2}=231$, *p* < 0.0001; figure 2*f*, Ca: diet × concentration, $x_{3}^{2}=92.1$, *p* < 0.0001; figure 2*h*, Mg: diet × concentration, $x_{3}^{2}=30.8$, *p* < 0.0001; 0 ppm treatment excluded from the analysis); also refer to electronic supplementary material, S4. Young workers offered Na diets consistently consumed more of the Na diets as the concentration increased; on average and on a daily basis, bees ingested more of the higher range of concentrations tested (500 and 1000 ppm) (figure 2*a*,*b*; electronic supplementary material, S5 and S6c,d). Interestingly, bees consumed more of Na diets at 500 ppm each day (electronic supplementary material, S6c), but bees presented with high Na diets (1000 ppm) showed a different pattern of feeding. We observed an increase in consumption of high Na diets after the first 24 h and until day 5, which was followed by an increase in consumption of the control solution instead (electronic supplementary material, S6d). Altogether, bees fed with the Na diets ate significantly more food (e.g. Na, 500 ppm: 58.5 µl bee^−1^) compared with control treatments (Na, 0 ppm: 47.8 µl bee^−1^). By contrast, the bees did not show clear preferences for the other salt-laced sucrose diets compared with sucrose alone (PI ≈ 0). For example, bees given diets containing K (10, 100 and 1000 ppm) or Ca (1, 10, 50 ppm) neither preferred nor rejected these diets, and consumed on average similar volumes from both control and test diets (figure 2*c*–*f*). However, the bees avoided consuming diets high in K (10 000 ppm), Ca (500 ppm) and in Mg (3000 ppm) (figure 2*c*–*h*); high Mg induced the maximum rejection observed for salts (PI < 0) (figure 2*g*,*h*). Analysis of the effects of salt diets on total food consumption revealed that the total volume ingested was also significantly affected by salt identity and concentration (GzLM: salt ×concentration, $x_{12}^{2}=88.5$, *p* < 0.001); refer to electronic supplementary material, S7 and S8). For the K and Ca treatments, the total diet consumed was not statistically different from the control. Bees fed the low-Mg treatments (10 and 30 ppm) consumed greater total diet volumes than control bees (electronic supplementary material, S8d). Though bees consumed less of high Mg diets (3000 ppm), they consumed more of the control solution alone. Thus, there was no significant impact on total food consumption; total amounts of food consumed between 0 and 3000 ppm were not statistically different (electronic supplementary material, S8d).

#### (ii) Metals

**Figure 3.**
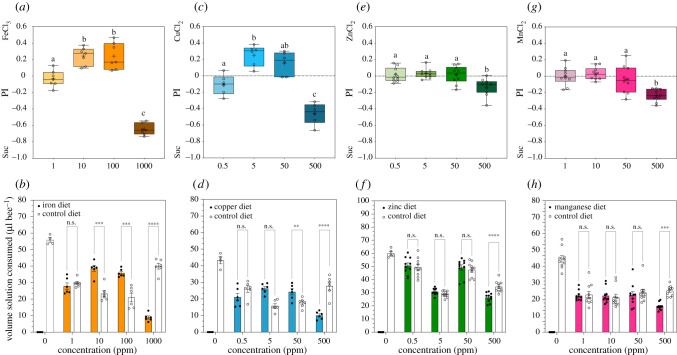
Consumption preference for metal-laced sucrose solutions in two-choice assays. (*a*,*b*) Iron, (*c*,*d*) copper, (*e*,*f*) zinc and (*g*,*h*) manganese treatments. (*a*,*c*,*e*,*g*) Boxplots refer to total consumption preference (PI) and indicate the minimum and maximum ranges for each independent mineral treatment; +, mean; −, median. Preference for the metal (PI > 0) or control (Suc, sucrose alone, PI < 0) diets. We performed one-way GzLM to test the effects of concentration for each independent mineral group. Preference for metals, (*a*) Fe: $x_{3}^{2}=557$, *p* < 0.001; (*c*) Cu: $x_{3}^{2}=133$, *p* < 0.001; (*e*) Zn: $x_{3}^{2}=22.2$, *p* < 0.001; (*g*) Mn: $x_{3}^{2}=79.6$, *p* < 0.001. Statistical differences between treatments are denoted by different letters (pairwise at *p*-value < 0.05). (*b*,*d*,*f*,*h*) Barplots indicate the measured volumes consumed from both solutions offered to bees (metal-laced and control diets); error bars represent s.e.m. We performed multiple comparison analysis to compare consumed volumes from both solutions at each level of mineral concentration; refer to electronic supplementary material, S4. Asterisks denote statistical significance at *p < 0.05, **p < 0.01, ***p < 0.001, ****p < 0.0001; n.s., not significant. Control treatment (sucrose-only diets, 0 ppm) was excluded from the pairwise comparison analysis. (Online version in colour.)

Feeding preferences and volumes consumed by young workers given metal-laced sucrose diets are shown in figure 3. Overall, preference for metal diets depended on the metal identity and its concentration (GzLM: metal × concentration, $x_{9}^{2}=265$, *p* < 0.001). For each metal treatment, the volumes consumed from each diet (control or test) depended on the diet and metal concentration (figure 3*b*, Fe: diet × concentration, $x_{3}^{2}=340$, *p* < 0.0001; figure 3*d*, Cu: diet × concentration, $x_{3}^{2}=104$, *p* < 0.0001; figure 3*f*, Zn: diet × concentration, $x_{3}^{2}=27.0$, *p* < 0.0001; figure 3*h*, Mn: diet × concentration, $x_{3}^{2}=28.0$, *p* < 0.0001; 0 ppm treatment excluded from the analysis); also refer to electronic supplementary material, S4. Young workers progressively ingested more of Fe-laced sucrose diets compared with sucrose control diet as the concentration increased; at low Fe (1 ppm) bees ate randomly (PI ≈ 0), i.e. similar volumes from both solutions; at 10 and 100 ppm bees showed maximum intake and preference (PI > 0) for these Fe diets (figure 3*a*,*b*). On a daily basis, consumption of Fe diets depended on the day and concentration (electronic supplementary material, S5, GEE: day × concentration, $x_{15}^{2}=123$, *p* < 0.001); at high Fe (1000 ppm) bees remarkably rejected Fe diets across the 6 day feeding period (PI < 0) (electronic supplementary material, S9d). Compared with control treatment, only bees offered high Fe (1000 ppm) ate significantly less food overall (electronic supplementary material, S7 and S10a). In a similar fashion, bees preferred to consume Cu diets at the medium range of concentrations tested (PI > 0 at 5 and 50 ppm); they ate more of the Cu-laced sucrose as the concentration increased (figure 3*c,d*) and for most of the days (electronic supplementary material, S5, GEE: day × concentration, $x_{15}^{2}=75.3$, *p* < 0.001; also see electronic supplementary material, S9f,g). On high-Cu treatments (0 ppm vs 500 ppm), bees notably preferred to consume the control diet. Surprisingly, it took these bees over the first 24 h to perceive and avoid ingesting high Cu diets (500 ppm, electronic supplementary material, S9h). Bees offered Cu diets consumed the least total volume of diet compared with total volumes ingested by bees in the control, Na, K, Ca, Mg, Fe, Zn and Mn treatments: 0 ppm (µl bee^−1^): Mn (67.0) > Ca (61.3) > Zn (60.0) > K (58.3) > Fe (55.8) > Mg (51.8) > Na (47.8) > Cu (43.3) (electronic supplementary material, and S8 and S10). Of note, bees in control treatments ingested between 40 and 65 µl bee^−1^ over the summer season (June to September); the total volume consumed by young workers was statistically different between mineral treatments and increased over time, except during the week we tested Cu treatments (electronic supplementary material, S11). Honeybees offered Zn or Mn treatments showed no preference nor rejection for diets containing 0.5–50 ppm of metal (figure 3*e*,*g*), and thus consumed similar volumes from both metal-laced andsucrose control diets (figure 3*f*,*h*); at high levels, bees rejected both Zn and Mn diets (both at 500 pm) and ate more of the sucrose-only solutions (figure 3*e*–*h*). Also, for both Zn and Mn, daily consumption depended on metal concentration only (electronic supplementary material, S5 and S9i–p). In metal treatments, the total volume consumed by bees from both solutions depended on the metal identity and its concentration (electronic supplementary material, S7 and S10). The total volume ingested by bees in Zn treatments was not linear, but oscillated across concentrations (electronic supplementary material, S10c). On average, bees in control, 5 or 500 ppm of Zn treatments consumed similar volumes of food (electronic supplementary material, S10c); instead, bees offered 0.5 or 50 ppm Zn diets ate similar volumes of food in total. By contrast, total food consumed by young workers in Mn treatments did not differ from control treatments (sucrose alone). Additionally, bees across Mn treatments ate, on average, larger volumes (≥ 60 µl) of food compared with all the other mineral treatments (electronic supplementary material, S10d and S11).

### (b) Water consumption, bee body weights and survival of young workers

#### (i) Salts

**Figure 4.**
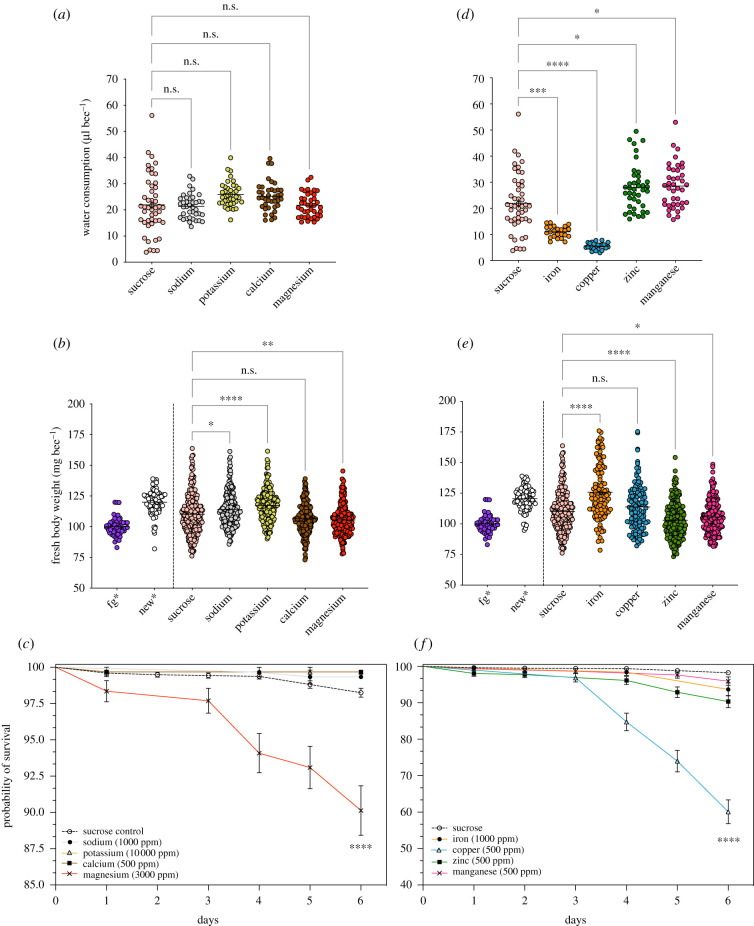
Summary data for water consumption, body weights and survival of bees offered mineral diets in choice feeding assays. Salts: (*a*) water consumed by bees fed sucrose-only, sodium, potassium, calcium or magnesium diets (pooled concentrations for each salt group, *n* = 39−48 boxes per treatment); Welch's ANOVA, *W*_4,100.3_ = 7.02, *p* < 0.0001 followed by multiple comparisons test (Dunnett's T3); (*b*) bee body weights (pooled concentrations for each salt group, *n* = 25–50 bees per treatment); Kruskal–Wallis, *H*(4) = 105, *p* < 0.0001, followed by multiple comparisons (Dunn's); (*c*) survival curves for bees fed high salt or sucrose-only (dashed line) diets. Metals: (*d*) water consumed by bees fed sucrose-only, iron, copper, zinc or manganese diets (pooled concentrations for each metal group, *n* = 24−48 boxes per treatment); Kruskal–Wallis, *H*(4) = 101, *p* < 0.0001, followed by multiple comparisons test (Dunn's); (*e*) bee body weights (pooled concentrations for each metal group, *n* = 20–65 bees per treatment); Kruskal–Wallis, *H*(4) = 117, *p* < 0.0001, followed by Dunn's test for multiple comparisons; (*f*) survival curves for bees fed high-metal or sucrose-only (dashed line) diets. (*a*,*d*,*b*,*e*) Sucrose control treatments were pooled together from eight independent experiments, *n* = 48 boxes and *n* = 253 bees, respectively; untreated bees (no feeding), foragers (fg, *n* = 50) and newly emerged workers (new, *n* = 89) are depicted for visual comparison only. *, Groups that are statistically different (*p* < 0.05) from the reference group (sucrose treatment); n.s., not significant; error bars, mean ± s.e.m. For detailed data for each mineral group refer to electronic supplementary material, S12 (salts) and S15 (metals). (Online version in colour.)

In figure 4*a*, we show the pooled data for water consumption from each salt group compared with the water consumed by control bees (data shows eight pooled treatments, Suc: 22.0 ± 1.58 µl bee^−1^ day^−1^), which were fed sucrose diets only. Overall, water intake was dependent on the salt treatment (GzLM: salt, $x{}_{4}^{2}=29.4$, *p* < 0.001). Within each salt group, the volume of water consumed by bees depended on both salt and concentration (GzLM: salt × concentration, $x_{12}^{2}=33.2$, *p* < 0.001; mean µl bee^−1^ day^−1^, Na: 20.6 ± 0.66; K: 25.6 ± 0.70; Ca: 25.3 ± 0.86; Mg: 23.1 ± 1.12); see electronic supplementary material, S12. Specifically, in the Mg group, control bees consumed on average more water than bees on Mg diets (GzLM: $x_{4}^{2}=24.0$, *p* < 0.001, electronic supplementary material, S12j).

At the end of each experiment, we weighed a subset of bees (*n* = 20–50 bees per treatment) to test the effect of mineral feeding on their total body weight. In figure 4*b*, we show the pooled data for bee body weights for all control treatments and for each salt group. Overall, dietary treatment had a significant effect on bee body weights at day 6 (GzLM: $x_{4}^{2}=124$, *p* < 0.001). The body weights of bees given Na (114 ± 0.89 mg bee^−1^), K (117 ± 0.87 mg bee^−1^) or Mg (105 ± 0.81 mg bee^−1^) diets were significantly differtent from control bees (110 ± 1.02 mg bee^−1^) (figure 4*b*). Within each salt group, there was a significant effect of salt type and concentration on the body weight of caged young workers (GzLM: salt × concentration, $x_{12}^{2}=52.4$, *p* < 0.0001). Within the K group, bees offered high-K diets had statistically higher body weights at day 6 compared with control bees (electronic supplementary material, S12e).

We then tested whether mineral feeding affected the mortality rates of caged young workers kept in a choice feeding context during 6 days. We used regression models to test the risk effect of concentration on bee survival for each salt group (electronic supplementary material, S12); we checked the proportionality of hazards assumption, i.e. the risk factors affecting time to event (death), which were constant over time for salt treatments. Overall, the lower range of mineral concentrations did not impact the survival of bees (electronic supplementary material, S12) compared with the control treatment. Therefore, we show the survival curves for high-salt feeding in figure 4*c*. The risk of mortality to bees depended on time and salt identity (Cox regression (reg.): salt, time × salt, Wald $x_{4}^{2}=44.7$, *p* < 0.001). Only high-Mg diets were statistically different from the control group, inducing around 10% of bee deaths by day 6 (figure 4*c*, Cox reg.: time × high Mg, Wald $x_{1}^{2}=32.3$, *p* < 0.0001, exp(*β*) = 1.38, 95% CI [1.23, 1.54]; electronic supplementary material, S12 and S13, Cox reg.: high Mg, Wald $x_{1}^{2}=8.59$, *p* = 0.01, exp(*β*) = 5.17, 95% CI [1.58, 16.9]). In decreasing order, the mean survival rates for high-salt diets were Ca = K (99.7%) > Na (99.3%) > Mg (86.8%) (figure 4*c*, electronic supplementary material, S14). At this range of concentrations (1–10 000 ppm), the salt diets did not have a negative impact on the survival of bees over 6 days of feeding. At this range of concentrations (1–10 000 ppm), the salt diets did not have a negative impact on the survival of bees. At the highest level tested, three (Na, K and Ca) out of four salt treatments still supported a high rate of survival by day 6 (electronic supplementary material, S12 and S14). Interestingly, K diets as high as 10 000 ppm (1% w/v) did not increase the risk of death compared with control treatments. More than 98.0% of bees were still alive in the K treatments by day 6 (electronic supplementary material, S12 and S14).

#### (ii) Metals

We show the pooled data for water consumption from each metal group in figure 4*d*. Water intake in metal treatments was dependent on the metal identity compared with the control treatment (GzLM: metal, $x_{4}^{2}=672$, *p* < 0.0001). Specifically, bees fed Fe (10.7 ± 0.41 µl bee^−1^ day^−1^) or Cu (5.36 ± 0.24 µl bee^−1^ day^−1^) diets consumed on average less water than all control bees (22.0 ± 1.59 µl bee^−1^ day^−1^). Within each metal group, water intake varied as a function of metal type and concentration (electronic supplementary material, S15; GzLM: metal × concentration, $x_{12}^{2}=104$, *p* < 0.0001). In fact, water ingestion significantly increased with concentration in Fe and Cu treatments, with high metals promoting the highest water consumption (1000 ppm of Fe: 13.4 ± 0.49; 500 ppm of Cu: 6.31 ± 0.65 µl bee^−1^ day^−1^) (electronic supplementary material, S15). By contrast, bees on Zn (28.4 ± 1.29 µl bee^−1^ day^−1^) or Mn (27.1 ± 1.17 µl bee^−1^ day^−1^) diets consumed more water, on average, compared with all control bees (figure 4*d*).

In figure 4*e*, we show the pooled data for bee body weights from each metal group tested. There was a significant effect of dietary metal on the body weight of caged worker bees compared with all control bees (GzLM: $x_{4}^{2}=125$, *p* < 0.0001). Body weights of bees given Fe (125 ± 1.82 mg bee^−1^), Zn (103 ± 0.98 mg bee^−1^) or Mn (107 ± 0.84 mg bee^−1^) diets were statistically different from control bees (110 ± 1.02 mg bee^−1^). Within each metal group, the body weights of bees depended on both metal and concentration (GzLM: metal × concentration, $x_{12}^{2}=50.2$, *p* < 0.001). Specifically, only bees fed Cu 50 ppm diets reported weights statistically different from the control bees (electronic supplementary material, S15e). No other metal treatments influenced bee body weight compared with control bees within each experiment (electronic supplementary material, S15).

Bee survival depended on the type of high metal and covaried with time (Cox reg.: time × metal, Wald $x_{4}^{2}=14.5$, *p* = 0.006). The risk was greatest for bees ingesting high-Cu diets (500 ppm) compared with all control bees (Cox reg.: time × high Cu, $x_{1}^{2}=10.2$, *p* = 0.001, exp(*β*) = 1.52, 95% CI [1.18, 1.97]; figure 4*f*). In decreasing order, the mean survival rates for high-metal diets were Mn (95.9%) > Fe (93.7%) > Zn (90.3%) > Cu (58.7%) (electronic supplementary material, S13–S15). Within each metal group, only Cu or Zn concentrations had a statistically significant effect on bee survival compared with the reference group (sucrose control bees) (Cox reg.: Cu diets, $x_{4}^{2}=11.5$, *p* < 0.001; Zn diets, $x_{4}^{2}=20.8$, *p* < 0.001; see electronic supplementary material, S13–S15). Individually, high-Cu or high-Zn diets induced the highest bee mortality compared with control and lower-range metal diets (Cox reg.: high Cu, $x_{1}^{2}=17.6$, *p* < 0.001, exp(*β*) = 68.0, 95% CI [9.47, 488]; high Zn, $x_{1}^{2}=6.81$, *p* = 0.01, exp(*β*) = 4.86, 95% CI [1.48, 15.9]). Unexpectedly, there was no significant effect of concentration on the survival of bees fed Fe or Mn diets (electronic supplementary material, S13 and S14). To assess whether late season had an impact on the survival of bees, we compared survival curves between control treatments (electronic supplementary material, S11c). Overall, experiments conducted later in the season (e.g. Mn) did not seem to have an impact on young workers' survival as there was no statistical significance between survival curves of control treatments (sucrose-only diet); more than 95% of bees were still alive by day 6 (electronic supplementary material, S11c). In decreasing order, the survival probabilities for control treatments across salt/metal groups were Ca = Cu = K (99.7%) > Fe (98.4%) > Mg (98.0%) > Zn (97.9%) > Na (96.6%) > Mn (95.4%) (electronic supplementary material, S13 and S14).

## 4. Discussion

Micronutrient intake is regulated via the coordination of pre- and post-ingestive processes that integrate the taste system with information about nutritional state. If bees in our study did not regulate the ingestion of minerals, they would have fed randomly and displayed no clear pattern of preference (i.e. equal consumption from both diets). Our data demonstrate that bees, like other animals: (1) are able to self-select mineral-enriched over mineral-free diets in a concentration-dependent fashion; (2) actively regulate their intake of Na, Fe and Cu; and (3) adjust feeding behaviour to avoid high and potentially toxic concentrations of seven out of eight of these micronutrients. We based our range of concentrations on previous recommendations for ash inclusion in synthetic diets for bees [54,75] and preliminary work in the laboratory [76]. It was surprising that few of the micronutrients we tested were phagostimulatory at low concentrations. Instead, the intake of K, Ca, Mg, Zn and Mn was regulated mainly by the avoidance of high concentrations.

Active regulation was clearly seen through the dietary selection behaviour of the bees in this study. At lower concentrations both the Fe and Cu diets stimulated feeding until a threshold (and maximum intake) was reached at 10–100 ppm (179–1790 µM Fe^3+^) and 5–50 ppm (78.7–787 µM Cu^2+^), respectively. Beyond these concentrations, high-Fe (1000 ppm) and high-Cu (500 ppm) diets became distastetul and induced a decline in both consumption and survival. In fact, high concentrations of Fe deterred feeding with an effect size greater than all the other mineral treatments. The nonlinear relationship between Na/Fe/Cu concentration and food consumption aligns with the Bertrand's rule prediction. It suggests that the optimal range for bee performance, ingestion behaviour and survival is near the range of concentrations most preferred. Interestingly, other insects exhibit active regulation of Fe and Cu. For example, in *Drosophila*, larvae and adults prefer to feed on diets relatively low in Fe (less than 30 mM; 1680 ppm Fe^2+^) and Cu (1 mM; 64 ppm Cu^2+^) while avoiding those with high concentrations (Fe^2+^: 40–70 mM; 2234–2910 ppm; Cu^2+^: 20 mM; 1271 ppm) [50].

We also found that honeybees actively regulated their intake of NaCl. Salt intake has been demonstrated in locust nymphs (as Wesson's salt mixture) and rats (as NaCl), where increasing concentrations of salt favoured consumption and fitness while concentrated solutions were avoided [44]. Trumper & Simpson found that locust nymphs regulated the intake of micronutrients independently of macronutrients around an optimal concentration of 1.8% of the salt mixture (dry weight, 900 ppm Na^+^) in an otherwise complete diet, but only when offered a choice [44]. At low levels of salt, locusts were less efficient at converting ingested food into growth; likewise, high-salt diets were avoided, reflecting their toxicity [44].

Simpson and Raubenheimer's models on nutrition largely assume that Bertrand's rule is the base model for the regulation of nutrients; in other words, low concentrations are phagostimulatory, whereas high concentrations are repellent or avoided [70,77]. Indeed, many of their studies have shown that macronutrients and micronutrients are regulated in this way. What is surprising is that we find that the majority of salts and metals we tested on honeybees are not. We found that all of the compounds we examined were avoided at high concentrations. This indicates that the bees are likely to use some form of post-ingestive feedback to regulate the intake of high concentrations. Alternatively, it could be the case that preferences for some salts (e.g. K, Ca, Mg) would be revealed only later in the season; we tested the salt diets during the peak of summer. Though, we did not test specifically for this, we observed variation in sucrose solution consumption over the season (electronic supplementary material, S11). Previous work showed that honeybee preferences for minerals in water vary during the season. [57,59,60] The fact that the bees did not prefer low concentrations of most of these dietary minerals, however, indicates that they probably lack the ability to taste most of these compounds at low concentrations.

One mechanism that bees could use to overcome toxicity is adjusting water intake. Drinking more water would allow them to dilute down concentrated solutions and prevent intoxication or other detrimental effects on health. Bonoan *et al.* found that foraging preferences for water solutions containing 1% of NaCl and MgCl_2_ tracked the variation in pollen [57,59]. Lau & Nieh, using harnessed honeybees, reported similar preferences for NaCl, KCl and MgCl_2_ at 0.1–1.5% in water solutions [78]. In our study, bees rejected high mineral concentrations in sucrose solutions (with the exception of high Na), but only high Fe and Cu increased total volume of water intake compared with control cohorts (electronic supplementary material, S15).

In the table (electronic supplementary material, S16), we compare our predictions for the preferred ranges for the eight minerals we tested against what is found in adult worker bees, pollen and honey in previous reports. All of our estimated preferred ranges fall within the values that were previously found in bees, particularly for Na (500–1500 ppm), Fe (10–200 ppm) and Cu (5–50 ppm). For all the remaining minerals, we only observed a rejection threshold, which is above the levels found in adult workers. The exception was K. Potassium levels reported in bees and pollen exceeded our threshold for deterrance (10 000 ppm). In fact, potassium has been reported to be up to 4× greater in some plant species' pollen than the concentration we found deterrent to honeybees (electronic supplementary material, S16). It was also surprising that bees in our study did not prefer 1000 ppm of K diets over sucrose alone. Honeybees have been reported to prefer 1500 ppm of K in sucrose solutions compared with sucrose alone, but ultimately these authors found that acceptance–rejection concentrations of nectar minerals are species- and concentration-dependent for K [63]. Similarly, bees were only deterred at high levels (500 ppm) of Zn and Mn diets. In honeybee colonies, however, sucrose solutions containing Zn (30–75 ppm) improved the antioxidant status, survival and brood-rearing capabilities of these colonies [36]. As for Mn, we hypothesize that this metal may not be as nutritionally relevant for adult workers as all the other metals. In fact, Zn and Mn seem to have a ‘sparing' effect, meaning they can replace one another in different metalloenzymes [79].

Overall, our work showed that young adult honeybees can actively regulate only three of the inorganic micronutrients we studied over the whole of their dynamic range in food. By screening each mineral, our study is the first to identify whether bees possess specific mechanisms particular to individual micronutrients. Bees can use taste to detect certain micronutrients—but not all of them. Post-ingestive processes can adjust feeding where taste cannot detect these micronutrients. This study along with others [11,56,80] paves new ground of information for future research in mineral nutrition and feeding behaviour of insect pollinators.

## Data Availability

Supplementary materials and data supporting the findings are provided as electronic supplementary material [81].
